# Network separation modeling and quantum computing for developing wildfire fuelbreak strategy

**DOI:** 10.1038/s44172-026-00585-9

**Published:** 2026-01-19

**Authors:** Samuel Dent, Kelsey Stoddard, Madison Smith, Andrew Strelzoff, Christopher Cummings, Jeffrey Cegan, Igor Linkov

**Affiliations:** 1 US Army Engineer Research and Development Center, Information Technology Laboratory, Vicksburg, MS USA; 2 US Army Engineer Research and Development Center, Environmental Laboratory, Vicksburg, MS USA; 3 Credere Associates, LLC, Westbrook, ME USA

**Keywords:** Natural hazards, Forestry, Engineering

## Abstract

Fuelbreak placement is an important consideration in fire management. Historically, strategies for placing fuelbreaks have fallen on the experience of fire managers such as by following ridgelines, and recent searches for a formal placement strategy have struggled to scale to large areas. Here we present a basic strategy utilizing equal graph partitioning and quantum computing to efficiently determine placements. By posing partitioning as a quadratic constrained binary optimization problem, D-Wave’s hybrid quantum optimization tool could complete the task in seconds. Results for the examined area show two alternatives to the ridgeline method in a so-called worst-case fire scenario: one with 2.9% improvement in land separation equality while clearing 76 less acres, and another with a 12.4% improvement by clearing 19 more acres. In a selected subsection, D-Wave’s hybrid solver performed faster than the SCIP solver but slower than the CPLEX solver, with the prospect for increased speed-up on larger problems. These findings demonstrate the effectiveness of equal graph partitioning for fuelbreak placement and the potential of D-Wave’s hybrid solvers.

## Abstract (web summary)

Fuelbreaks are treated areas of land that mitigate the spread of wildfires. Dent and colleagues present a method for determining optimal placement using network science and partitioning through optimization with the D-Wave hybrid quantum solver.

## Introduction

In California, 15 out of 20 fires on both lists of the most destructive CA wildfires and the largest CA wildfires have occurred since 2010^1^. With incidents such as these in recent years, wildfire mitigation and fire management methods have never been more important. A common method by which forest managers combat these fires is through fuelbreaks^2^. While firebreaks are generally created in emergency situations to stop the spread of wildfire by removing fuels such as vegetation down to the bare soil, fuelbreaks are more long-standing, treated areas of land created ahead of time that lower, but do not eliminate, the risk of wildfires spreading^3^. Current approaches to deciding where to put fuelbreaks take into consideration a wide range of criteria, including the (i) fire environment, such as fuels, terrain, and fire weather, (ii) environmental features, such as previously burned areas, rivers, croplands, and grasslands, which may be suitable to connect fuelbreaks, (iii) terrain, such as ridgelines or valley bottoms in steep regions, and (iv) implementation criteria, such as accessibility and firefighter safety^2^. When designing and placing these fuelbreaks, it is critical to their effectiveness and to community safety to keep these objectives in mind^4,5^. However, upfront and maintenance costs will also be of crucial importance, especially for large areas of land. While these are important considerations, it begs the question - How can we determine the optimal locations for fuelbreaks?

Historically, methods to determine fuelbreak locations have been ad hoc by those managing fires. In recent years, however, there has been a growing interest in using analytical tools to achieve better strategies for where to place these breaks. For example, one study uses a greedy implementation of Dijkstra’s algorithm to determine an optimal location for fuelbreaks^6^. Another study utilizes a pre-established burn probability model, Burn-P3, in order to determine where best to place these breaks^7^. Due to the interconnected nature of wildfires, network science has increasingly become a promising approach to this problem. For example, one study uses a *k*-graph partitioning approach to determine a subset of links between nodes to cut, finding the optimal value using a genetic algorithm^8^. One limitation here is that the model is tested only on hypothetical examples, rather than real forest environments. A similar study employed a network approach to optimize the location of preventative measures like prescribed burns and thinning^9^. A common challenge in network science approaches, as highlighted in these papers, is the substantial computational difficulty involved in finding an optimal solution^8^.

More broadly, this approach to determine optimal fuelbreak locations fits into a class of similar problems characterized by networks that are partitioned into *k*-disjoint subsets, and there is ample literature examining how to fragment a network with a minimum number of node removals^10–12^. Similar network science disruption frameworks have been applied in a variety of fields including immunology, cybersecurity, and information theory to model ways to effectively curb propagation of some subject-dependent entity that spreads^13–15^. The aim of these efforts is to understand how best to minimize spreading within a network given its problem-specific topology.

In this paper, we draw on parallels between these spreading phenomena and the spreading in wildfires to examine how robustly network fragmentation can be used to effectively determine optimal locations for fuelbreaks within forest systems. In particular, we generated a forest network based on real data to simulate fire propagation and framed the problem as a variation of equal graph partitioning. Using binary constrained optimization on D-Wave’s Constrained Quadratic Model (CQM) hybrid quantum optimization tool, which we will refer to as the D-Wave hybrid solver, we found two representative solutions that offer alternatives to the traditional ridgeline method in a worst-case fire scenario (described in the Methods section). One solution had a 2.9% improvement in land distribution equality while using 76 less acres compared to the ridgeline method, and the other presented a 12.4% improvement by treating 19 more acres. The performance of D-Wave’s hybrid solver was also compared to the open-source SCIP solver and the commercial CPLEX solver on a smaller subsection of the network. All three solvers produced sufficient solutions, with CPLEX solving the fastest at ~0.5 s, D-Wave the next quickest at roughly 10 s, and the SCIP solver finishing last at over 10,000 s. More research is needed for comparing the solvers’ performance as the hybrid solver is expected to outperform classical solvers on much larger problems than the one presented in this paper. However, the results of this work reveal important considerations for determining where to place fuelbreaks, and the cross-domain nature of this work means that it contributes to the literature in multiple fields. Specifically, the work contributes to wildfire mitigation researchers seeking more systematic strategies for fire prevention, introducing a structured, optimization-based approach to wildfire fuelbreak placement. Network partitioning has been explored in fields such as epidemiology and cybersecurity, and here we apply it to firebreak optimization in conjunction with quantum computing. Additionally, it contributes to the work of optimization scientists exploring network separation problems in real-world scenarios. In particular, it presents a method to construct simple fire-propagation networks and demonstrates how network optimization and quantum computing can enhance large-scale environmental decision-making. Finally, it is relevant to quantum computing research investigating the advantages of annealing-based solvers for large-scale environmental problems. By framing fuelbreak placement as a constrained optimization problem, we demonstrate the potential for advanced computational techniques to enhance wildfire management and open new avenues for interdisciplinary research. While its impact on optimization and quantum computing is incremental, it presents a practical application of quantum-enhanced optimization in fire management, offering a foundation for future refinements and benchmarking against alternative methods.

## Methods

The methods employed in this paper can be separated into two efforts: those used to construct the fire propagation network, and those used to optimally separate that network. The first effort focuses on abstracting real-world forest topology and fire spreading models to a simplified network model. The second effort focuses on applying algorithms to find optimal, or semi-optimal, separation groups. A key component of this research question is where fuelbreaks can be placed in an ecosystem. However, challenging this network construction and its optimized separation are the real-world practicalities and desires to reduce the amount of land dedicated to fuelbreaks. Large, cleared areas can disrupt ecosystems, displace wildlife, and limit land use for recreation and conservation. Therefore, achieving effective separation with the minimal disturbance to the network is ideal—ensuring that fuelbreaks are strategically placed to maximize impact while minimizing ecological and social footprints which can support more enduring and socially acceptable wildfire management practices.

The scenario under consideration is a worst-case where (i) no information about the fire is known beforehand, e.g. where the fire starts, (ii) the fire is assumed to spread wherever possible, consuming all parts of the forest connected to the fire origin, and (iii) we can only place fuelbreaks ahead of time, which we consider 100% effective at preventing fire from spreading at that location.

### Fire propagation network construction

For this problem, we formalized an approach by which we can represent fire propagation risk as a network of spatially related entities. This network should mimic other previously studied spreading networks. An example is infection networks, which are often constructed from social networks, where nodes represent people or potential hosts of an infection and edges connect people who are exposed to each other, creating a potential opportunity for the spread of disease^16^. Similarly, for the case of a fire spread network, the nodes represent the locations at which fire can be present, and edges connect the locations between which fires can spread.

**Fig. 1:**
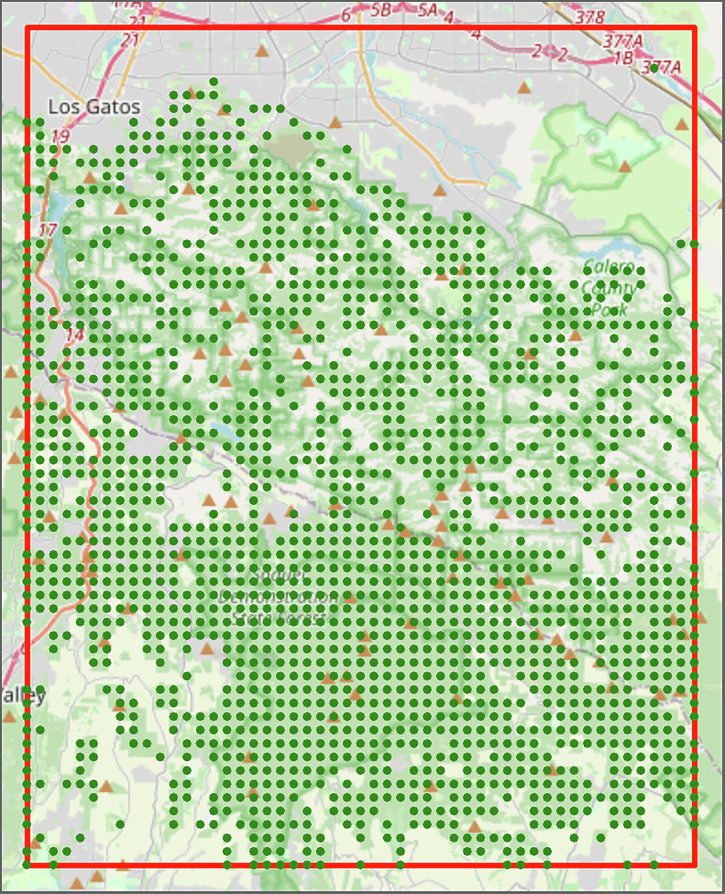
Forest grid in region of interest (ROI). Shows the points from the grid of 51 by 63 which lie within the forested portions of the defined ROI [121.75° West – 122° West] and [37° North – 37.25° North]. These points are the potential locations for the nodes in the fire propagation network before removing isolated subgraphs/subnetworks.

To develop such a network, we first determine the nodes. This starts by defining a region of interest (ROI), which we defined to be between [121.75° West – 122° West] and [37° North – 37.25° North] in California. The forested areas in that ROI are then identified by querying OpenStreetMaps^17^, defined to be regions with either the ‘landuse’ tag set to ‘forest’ or the ‘natural’ tag set to ‘wood’. To discretize these areas into nodes, a grid of 51 by 63 points is defined over the area of interest. This grid size was selected so the *x* and *y* spacing between points is approximately equal and so that the network produced is densely, but not overly, connected. Other grid dimensions may be chosen based on the ROI. Grid points which lie within the forested areas are then kept for consideration as network nodes. Both the forested areas used and the filtered set of points are shown in Fig. 1. The elevation of each point was queried via the Elevation Point Query Service^18^ and stored as a variable to be used in the edge creation steps.

Next, we define the network edges. There are several ways in which a fire propagates, including spreading, short-range spotting, and long-range spotting, as described by the National Wildfire Coordinating Group (NWCG)^19^. Spotting describes when embers or firebrands are carried away from the original source of the fire, and land on additional fuel to start a new ‘spot’ fire. Long-range spotting occurs when the firebrands are lofted into the air and travel a considerable distance before landing. The maximum on-ground distance those firebrands can travel, while remaining lit to be able to start new fires, is called the maximum spotting distance. Short-range spotting describes spotting at distances less than the maximum spotting distance and is often omitted from fire modeling on the assumption that this type of spotting is typically accounted for when modeling fire spreading. Therefore, only long-range spotting and spreading are typically accounted for in the creation of this type of network. For simplicity, only long-range spotting was considered in modeling the fire propagation for this paper. It should be noted that real fire propagation is influenced by the previously mentioned factors, such as environment and terrain, which could be incorporated into the edge creation process described below in future studies.

Nodes that can spread fire between each other are connected via an edge in our network structure. To account for long-range spotting, an edge is created between two nodes if the distance between them is less than the maximum spotting distance, given by:

1 $$S= 	 \, \left(-1.253\times {\,10}^{-4}\,W+3.304\times {10}^{-5}\right) \\ 	 \times \left(T+E\right)+0.4297\,W+0.01065$$

where *S* is the maximum fire spotting distance in miles, *W* is the wind speed in mph, *T* is the tree height in feet, and *E* is the elevation difference between the points in feet. This equation was derived from the “NOM 4. Maximum Spotting Distance (Maximum Spotting Distance)”^19^ chart provided by the NWCG. In this equation, it is assumed that the average tree height is 50 feet and that there is 40 mph wind which can be coming from any direction. In addition to simplifying the network creation process, accounting for all wind directions aligns with the worst-case scenario where no prior information about the fire or wind is available. The resulting network contained multiple small isolated subgraphs, e.g. the cluster of forest nodes in the southwest corner of Fig. 1. These small isolated subgraphs would not be impacted by fire spreading in the main body of the network but would affect the optimization. Figure 2 shows an example fire propagation network without isolated subgraphs. After removing the isolates, the resulting network retained a total of 1492 nodes and 4637 edges.

**Fig. 2:**
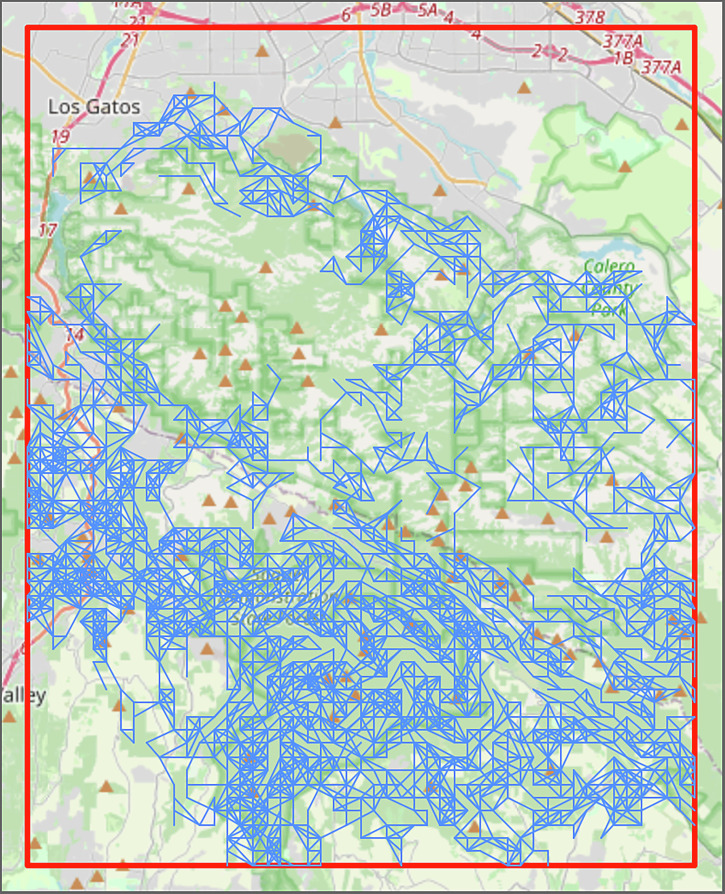
Fire propagation network in region of interest (ROI). Shows the edges in a fire propagation network in the ROI [121.75° West – 122° West] and [37° North – 37.25° North]. These links represent the determined vectors along which fire is able to spread based on the maximum spotting distance calculations from Eq. (1).

### Network separation technique

To separate this network, we draw on the work from the field of immunology^20^, which aims to solve this problem by separating the nodes of the network into three disjoint groups. Two of those groups are subnetworks which represent the parts of the forest which remain untouched, i.e. not converted to fuelbreaks, and the third is a separator group of nodes that represents the optimal location to place a fuelbreak. This approach is adopted because in both abstract and real social networks, it demonstrated good performance, and was able to separate a graph with 5–50% fewer removed nodes than previous methods such as high degree target (HD), high degree adaptive (HDA), and high betweenness (HB) target strategies^20^.

**Fig. 3:**
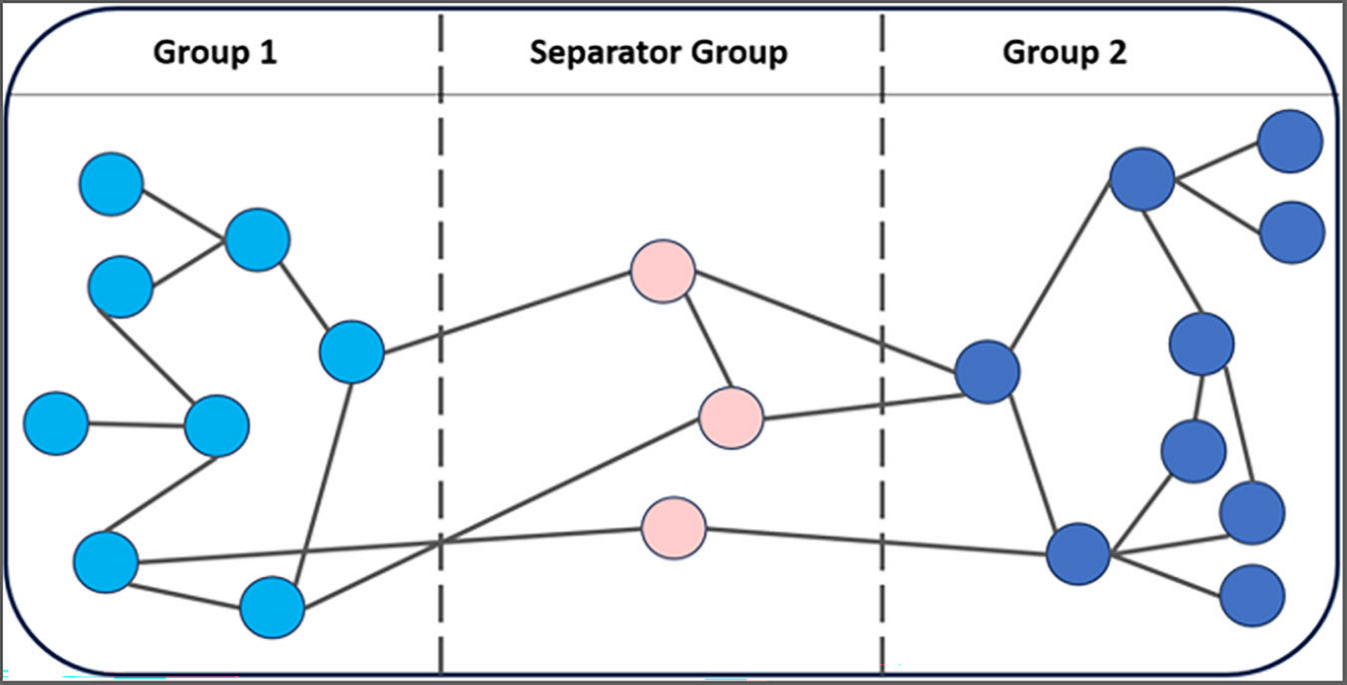
Example of a partitioned graph. Shows a representative example, demonstrating how the graph will be separated into three groups. Note here that while the aim is to separate the graph (i.e., assign the nodes so that there are no cross-edges directly between Group 1 and Group 2) such that Group 1 and Group 2 are of equal size, a tolerance has been added to allow the group sizes to vary by a specified amount. In this example, the tolerance is 1 with Group 1 containing 8 nodes and Group 2 containing 9 nodes.

In this formulation, the network is separated using equal graph partitioning (EGP), which aims to fragment a network into connected subnetworks of equal size. This fragmentation is done by assigning nodes to one of three groups: the two equally sized subnetworks and a third separator group. The separator group consists of the nodes that, if removed, should completely disconnect the other two subnetworks (Fig. 3). The aim is then to minimize the size of the separator group. EGP lends itself to our worst-case scenario since we do not know where the fire will begin. For instance, consider an unequal partition where 80% of the nodes is contained in Group 1 separated from Group 2 containing 20%. Assuming we have no additional information, the fire has an equal chance of starting at any node, so there is an 80% chance it will start in and consume Group 1 and a 20% chance for Group 2. That is, there is an 80% chance the fire will consume 80% of the forest with this group configuration. On the other hand, if the groups are equally sized, only 50% of the forest will be consumed. Thus, having equal group sizes reduces the chances of a larger portion of the forest being consumed.

Identifying the partitions is done by posing the problem as a Constrained Quadratic Model, where an objective function and constraints are defined. To formulate the model in line with D-Wave’s example^21^, we define a binary assignment variable*, x*_*i,j*_, which will be used to describe whether node *i* is in Group *j*:

2 $${x}_{i,j}=\left\{\begin{array}{l}1,\,{{\rm{if}}} \, {{\rm{node}}} \, i \, {{\rm{is}}} \, {{\rm{in}}} \, {{\rm{group}}} \, j\\ 0,\,{\mbox{otherwise}}\hfill\end{array}\right.$$

Here, Group 1 and Group 2 are the two partitioned groups, while Group 3 is the separator group. We will then set up the optimization to minimize the objective function:

3 $$\displaystyle{\sum}_{i=1}^{n}{x}_{i,3}$$

Equation (3) is therefore stating that the optimization is seeking to minimize the number of nodes that are assigned to the separator group, i.e. the number of fuelbreaks. This minimization is solved while being subject to the following constraints:

4 $$\displaystyle{\sum}_{j=1}^{3}{x}_{i,j}=1,\; {{\rm{for}}} \, i=1,2,\ldots ,n$$

5 $$\displaystyle{\sum}_{\left(a,b\right)\in E(G)}\left({x}_{a,1}{x}_{b,2}+{x}_{a,2}{x}_{b,1}\right)=0,\; {{\rm{where}}} \, E(G) \, {{\rm{is}}} \, {{\rm{the}}} \, {{\rm{set}}} \, {{\rm{of}}} \, {{\rm{graph}}} \, {{\rm{edges}}}$$

6 $$\displaystyle\left|{\sum}_{i=1}^{n}{x}_{i,1}-{\sum}_{i=1}^{n}{x}_{i,2}\right|\le t$$

Here, Eq. (4) adds the constraint that all nodes must be uniquely assigned to one of the three groups. Equation (5) states that there should be no cross-edges between Group 1 and Group 2, meaning fire cannot spread between the two. Finally, Eq. (6) imposes that Group 1 and Group 2 should be made approximately the same size, with a group size difference tolerance of *t*. This differs from strict EGP in that Eq. (6) allows near-equal groups, and the tolerance *t* can be changed as part of the experiment.

These equations are then solved using D-Wave’s hybrid solver for Constrained Quadratic Models (CQM). By hybrid, we mean it uses both quantum and classical computing techniques to approximate the global optimum to our problem. A hybrid approach allows exploitation of the benefits of quantum and classical computing on the subproblems they are most suited to solve efficiently. The problem can be decomposed into subproblems, and then these subproblems are allocated to either classical or quantum processing unit resources.

For the quantum computing method, D-Wave uses quantum annealing, which shares similarities with its classical counterpart, simulated annealing, although it is often more efficient^22,23^. One approach to solving the network separation problem is by sampling from so-called ‘low-energy’ states. The samples give us information about the model for a given set of parameters which we can manipulate in order to find the minimum of our objective function. Although a full discussion about the quantum annealing algorithm is beyond the scope of this paper, it is important to note that these sampling algorithms can be very computationally intensive, and quantum annealing can greatly improve the speed of this process^24^.

There are limitations to the above outlined quantum annealing process. As an approximate solver, it can be drawn to local minima rather than a global minimum, thereby providing sub-optimal solutions. Additional strategies can be employed to refine the solutions, such as running the solver multiple times for the same set of parameters or combining the results with those of classical methods like in ensemble averaging. Moreover, the qubits in the system are limited. In the fire network built above, the configuration of the nodes and edges does not directly match the topology of the qubits available in the solver, and methods such as couplers must be used to match the configuration, potentially adding complexity. Furthermore, since spins in a magnetic field are either up or down, they are suited to solve binary integer problems, whereas in our case we have three groups we want to separate the nodes into rather than just two. This is circumvented by having three binary variables, *x*_*i,1*_, *x*_*i,2*_, and *x*_*i,3*_, per node *i*, and with the one-hot constraint in Eq. (4), each node can only be in one group. However, this strategy also multiplies the number of variables required and makes the problem itself larger. For these reasons, it would be advantageous for a classical computer to cut up our original problem into a set of subproblems which the quantum computer is best suited to solve. D-Wave’s hybrid quantum computer is uniquely postured to solve problems like this, where the quantum and classical processing units can be used in their optimal domains to come to an overall solution that proves to be efficient and useful to real world scenarios.

To compare the performance of D-Wave’s quantum hybrid solver with classical solvers, a network separation problem was also run on IBM’s CPLEX solver and the SCIP solver in the Python package CVXPY. The Community Edition of CPLEX was used, which has a limit of 1000 variables, where there are roughly three times as many variables as nodes for the network separation problem. With this in mind, a piece of the California fire network that was small enough to satisfy this limitation was chosen: [121.75° West – 121.875° West] and [37° North – 37.058° North], containing 267 nodes. For simplicity, the optimization problem was set up with equal graph partitioning, where the group size difference tolerance is 1. For both of these classical exact solvers, a slight change to the optimization formulation is necessary. In particular, the cross-edge constraint of Eq. (5) is not convex, which poses issues for both solvers. For the CPLEX solver, we replace Eq. (5) with the constraints:

7 $${\left({x}_{a,1}+{x}_{b,2}\right)}^{2}+{\left({x}_{a,2}+{x}_{b,1}\right)}^{2}\le 2,\;{{\rm{for}}} \, {{\rm{all}}} \,\left(a,b\right)\in E(G).$$

Note that the case where edge (*a*,*b*) is a cross-edge is the only instance where the left-hand side of Eq. (7) should give a value greater than 2. The SCIP solver in CVXPY requires further changes, as it only accepts problems that follow the Disciplined Convex Programming (DCP) system. For the network separation problem, this effectively means that the constraints cannot contain quadratic terms, such as in Eqs. (5) and (7). To remedy this, we write the quadratic cross-edge constraint into a convex objective function, and enforce the number of separators as a constraint. Then we can write the SCIP solver’s objective as minimizing

8 $$\displaystyle{\sum}_{\left(a,b\right)\in E(G)}\left[{\left({x}_{a,1}+{x}_{b,2}\right)}^{2}+{\left({x}_{a,2}+{x}_{b,1}\right)}^{2}+{x}_{a,3}^{2}+{x}_{b,3}^{2}\right],$$

with a separator constraint

9 $$\displaystyle{\sum}_{i=1}^{n}{x}_{i,2}=s,$$

for some number *s*. The objective function of Eq. (8) has a similar form to Eq. (7) but includes terms related to the separator group so that all cases other than a cross-edge return the same value for the expression inside the summation, and only the cross-edge case is punished. Equation (9) requires prior knowledge of the number of separators, but since the SCIP solver is being used to compare against the D-Wave hybrid solver, we can run the hybrid solver or the CPLEX solver and use the number of separators from its solution for Eq. (9). For the smaller sub-network with 267 nodes, the value of *s* = 7 was used, and for the larger network with 1492 nodes, *s* = 11 was used.

## Results

The network used for the main experiments contained 1492 nodes and 4637 edges. We ran the constrained quadratic optimization problem on D-Wave’s hybrid quantum solver for different group size difference tolerances, recording the groups and separators found for each tolerance value.

### Network separation

**Fig. 4:**
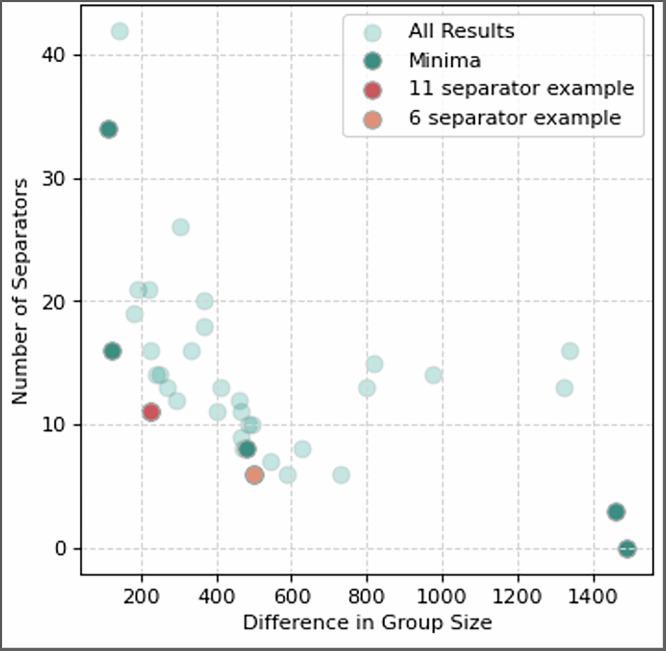
Number of separators vs the difference in group size. Shows the results of the fire spreading network separation using D-Wave’s quantum hybrid solver, comparing the number of separators in Group 3 against the difference in size between Group 1 and Group 2, related to the tolerance in Eq. (6). We see a general trend that allowing a larger difference in group size requires fewer separator nodes.

We varied the tolerance from 0 to 1500, using evolving step sizes roughly ranging from 20 to 200, applying larger step sizes as the tolerance increased. The number of nodes in the separator group as a function of the group size difference is shown in Fig. 4. Of note in this method is that sometimes a solution with higher group size difference and more separators than a previous solution will be identified as ‘optimal’ by the solver. This happens because the solver used is an annealer, an approximate solver rather than an exact one. So, it does not always find the global minima and instead sometimes identifies a local minimum. Additionally, the solver is a hybrid quantum-classical solver, which may experience quantum noise, possibly affecting the solutions found. To identify the best solutions, we labeled the solutions which have a higher group size difference but larger or equal number of separators to a previous solution as ‘All Results’ in Fig. 4. These represent the less-than-ideal solutions, possibly where the solver found a local minimum or experienced quantum noise.

**Fig. 5:**
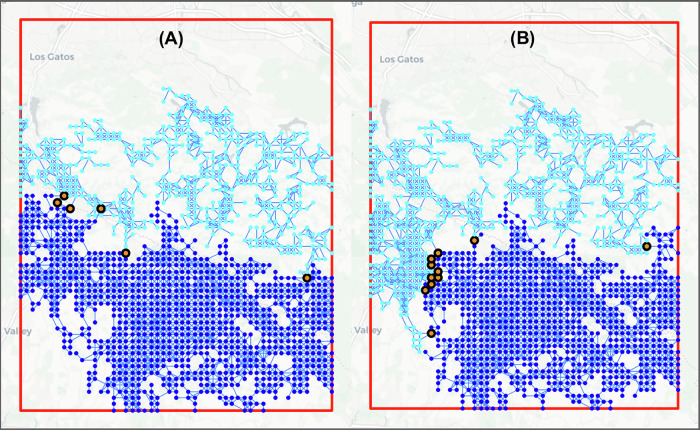
Example separated fire network. Shows a representative example of the results of the fire spreading network separation. Here, Group 1 and Group 2 are shown in light and dark blue, respectively. Group 3, the separator group, is shown in orange. **A** Shows the three groups when for the 11-separator solution. **B** Shows the groups for then 6-separator solution.

Two representative examples have been selected for consideration: among the solutions found for the various group size difference tolerances, one solution achieved graph separation using 6 separators and a group size difference of 500 while another achieved separation using 11 separators and a group size difference of 223, hereafter referred to as the 6-separator and 11-separator examples or solutions. In Fig. 4, those examples have been colored in red. The separated networks for these examples are shown in Fig. 5.

### Comparison with other methods

**Fig. 6:**
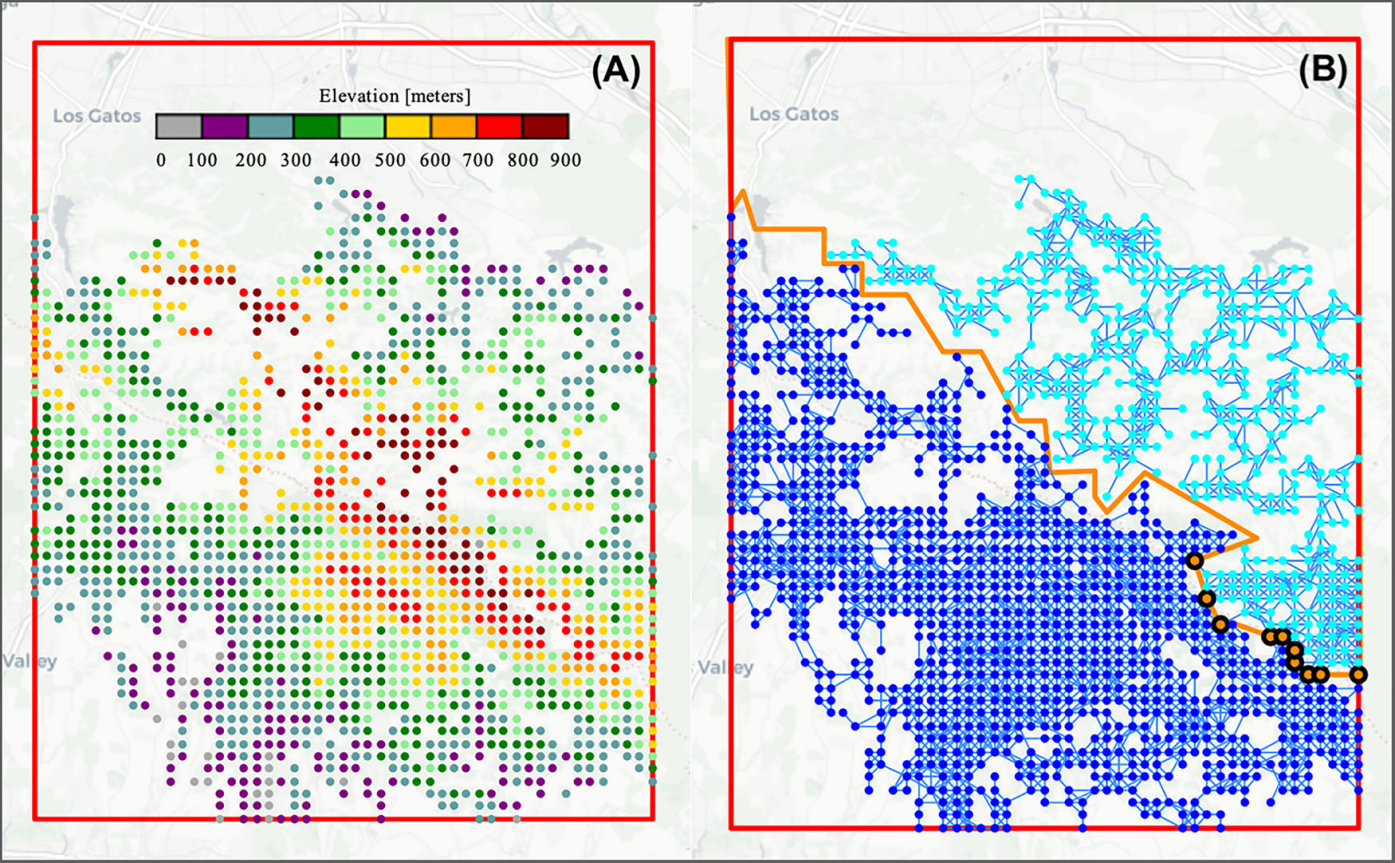
Separation of the fire propagation network based on the ridgeline. **A** Shows the nodes in the fire propagation network, colored by elevation. **B** Shows the results of the ridgeline separation, where the blue groups are Groups 1 and 2, and the orange group are the separator group. This group represents the locations of the fuelbreaks. The orange line shows the line of separation between Groups 1 and 2.

One approach used in practice to identify an effective location of fuelbreaks is simply to place the breaks where there is a ridgeline. To understand the quality of our approach, we compare our method with different numbers of separators to the more simplistic ridgeline separation approach. To do this, we examined the elevation of the nodes in the fire separation graph (Fig. 6A). Based on this elevation assessment, the smallest group of nodes which would separate the graph while following the highest ridgeline were determined (Fig. 6B), resulting in 10 separators. The number of nodes in each group using this method is then compared with the two representative examples from the graph separation of Fig. 6 in Table 1.

**Table 1:** Number of nodes in each group – CA network

Technique	Group 1 size	Group 2 size	Separator group
Graph separation − 11	852	629	11
Graph separation − 6	993	493	6
Ridgeline separation	1037	445	10

Of note is that the 11-separator and 6-separator solutions have been selected for a more detailed examination as they are the examples which provide the most direct comparison to the number of separators in the ridgeline method while providing high-quality solutions. In particular, we not only compare the objective of the number of separators found by the methods, but also the difference in the group sizes found. The number of separators is related to the amount of land that must be cleared for fuelbreaks, which we would like to keep small as to minimize the effect on the environment, while the group sizes correspond to how much area could potentially burn away in our worst-case fire scenario. So, we must balance these two land conservation concepts in our solutions. The 11-separator example is the solution which has the closest number of separators to the ridgeline solution. A comparison of the results for the two shows that for a similar level of removal, the largest group goes from having 69.5% of the nodes in the ridgeline solution to 57.1% of the nodes in the 11-separator example, making the separation much more equal.

The 6-separator example is also shown and has comparable group sizes to the ridgeline separation. This shows that using the graph separation optimization methodology achieves nearly the same quality of separation while only needing to remove 60% as many nodes. By dividing the total area considered by the number of nodes in the original grid, we found that each node covers an area of ~19 acres. Using this, Table 2 re-expresses the data in terms of the number of acres covered by each group.

**Table 2:** Number of acres covered by each group – CA network

Technique	Largest group area	Group area difference	Removal area
Graph separation – 11	16,160 acres	4230 acres	209 acres
Graph separation – 6	18,834 acres	9484 acres	114 acres
Ridgeline separation	19,669 acres	11,229 acres	190 acres

Here, it can be seen that the largest group area, by which we mean the largest side, of the ridgeline separator was 19,669 acres. This means that at most 19,669 acres are more likely to burn, in our scenario. Given the assumption that a fire can start anywhere with equal probability, the probability that the 19,669 acres would burn is ~70%. Comparing this with the graph separation solution with 6 separators, however, shows that an equally effective separation could be achieved removing only 114 acres. Here we are assuming for simplicity that all acres are equally easy and cost-effective to treat. Therefore, our method shows a 76-acre decrease in the overall area of fuelbreaks needed to be effective in limiting fire propagation through our modeled forest network. This is an important result of our methodology because of the maintenance involved in fire fuels management in these fuelbreak regions. This result is achieved with only 6 separators. Using the logic of our worst-case scenario and the 6-separator solution, there is a 66.6% chance that 18,834 acres will burn while the solution separates the graph more evenly and reduces the largest area that could burn by 835 acres (4.25%). In other words, not only does the 6-separator solution require 76 less acres to be removed, but it also improves the worst-case scenario fire possibility where the fire starts in and consumes the larger group area. Thus, graph separation shows itself to be a vast improvement in this case.

Comparing the 11-separator solution with the 6-separator example reveals a trade-off. The 11-separator solution requires clearing 209 acres, which is a 10% increase in the size of the fuelbreak compared to the ridgeline method. However, this option achieves a much more even separation of the network. In this scenario, the largest area that could potentially burn is reduced by 3509 acres (17.8%). While this markedly decreases the possible burn area, it also demands the largest removal area of the options considered. This highlights the challenge of balancing effective fuelbreak placement with the desire to minimize the amount of land dedicated to fuelbreaks.

### Quantum and classical comparison

**Fig. 7:**
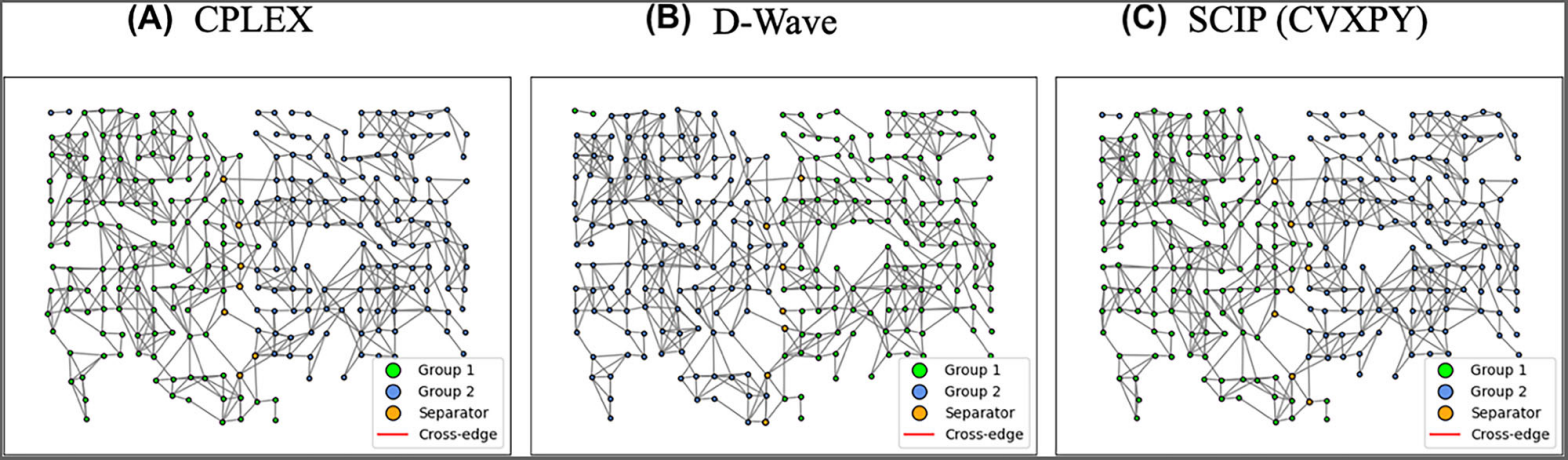
Quantum and classical results - visual comparison. The figure shows the group assignments produced by the **A** CPLEX, **B** D-Wave hybrid, and **C** SCIP solvers for the subset of the CA ROI. All three achieve separation without cross-edges, Groups 1 and 2 both having 130 nodes, and 7 separators. A small amount of random noise has been applied to the node positions in the visualization to make viewing the edges easier.

D-Wave’s hybrid solver was compared to the classical CPLEX and SCIP solvers on the subnetwork of 267 nodes. Each of the solvers produced an answer with 7 separators and Groups 1 and 2 both having 130 nodes. The node assignments from the solvers are visualized in Fig. 7. The solvers were timed, with the results shown in Table 3. We see that on this sub-network, the CPLEX solver completes the fastest in all stages, with the D-Wave hybrid solver exhibiting the next fastest completion time, and the SCIP solver finishing last. Additionally, while the CPLEX Community Edition solver could not run on the larger network, the D-Wave hybrid solver and the SCIP solver can. Both the 6-separator and 11-separator solutions take 5 s of solver time with the D-Wave hybrid solver, whereas the SCIP solver with CVXPY ran for 24 h without completing.

**Table 3:** Quantum and classical results – timing on CA sub-network

Solver	Build time (s)	Solver time (s)	Latency time (s)	Total time (s)
CPLEX	0.0692	0.4523	–	0.5215
D-Wave	0.1673	5.0000	4.3481	9.5154
SCIP (CVXPY)	0.1108	10,908.2753	–	10,908.3861

## Discussion

The decision of where to build fuelbreaks is complex and multifaceted, and ultimately dependent upon the specific geography, vegetation, and societal needs of the region under consideration. This paper addresses the question of fuelbreak placement by posing the problem as a network separation problem. In doing so, we add to the existing literature in three ways. First, in contrast to some previous research with wildfire networks^8^, we determine so-called separator groups to be used as fuelbreaks. A comparison of our approach with a ridgeline separation methodology shows that our approach provides a more efficient solution which requires fewer acres dedicated to fuelbreaks. Second, compared to the prior algorithmic approaches mentioned, we demonstrate how maximum spotting distance can be incorporated to construct the fire spreading network, which is a key variable in wildfire propagation. Third, a limitation of the approach is that node separation is a time-intensive algorithm, especially for large networks, making it impractical. For example, the exact solver SCIP in the CVXPY package takes over a day in runtime when attempting the network separation problem on the larger network of 1492 nodes. To address these issues, we explore the use of quantum computing, and quantum annealing specifically, to derive near-optimal separations more efficiently. In particular, compared to classical simulated annealing, quantum annealing has exhibited improved scalability for certain problem classes^25^, which is particularly important for our problem size. Our work demonstrates that quantum computing technology, which shows promise, can achieve fast convergence and provide effective solutions for network optimization problems.

The results of this study have several important implications for fire management policy and decision-making. Our findings demonstrate that optimization methods can more effectively separate forested land while requiring fewer interdiction points or fuelbreak constructions. Specifically, the optimization can achieve a 2.9% improvement in the equality of land separation while reducing the cleared area for fuelbreaks by 76 acres. Alternatively, increasing the cleared area by just 19 acres can result in a 12.4% improvement in equality. A key trade-off observed in the study is between the number of separators and the total area that needs to be removed. More separators allow for a more even partitioning of the network nodes into two groups but require a larger cleared area. This trade-off highlights the importance of balancing effective fuelbreak placement with the desire to minimize land disturbance. Expanding on these findings, fire managers can use network separation techniques to strategically place fuelbreaks, optimizing their impact while conserving as much land as possible. This approach can inform practical decision-making by providing a range of solutions with different numbers of separators, allowing managers to select the one that best fits their specific needs and priorities.

The broader policy implications are meaningful. Incorporating these optimization methods into national wildfire management strategies can enhance the efficiency and effectiveness of fuelbreak placement on a larger scale. This integration can lead to better resource allocation, reduced environmental impact, and improved protection of communities and natural ecosystems. By adopting these advanced techniques, fire management agencies can develop more resilient strategies to combat the increasing threat of wildfires, ultimately contributing to more lasting and effective wildfire management practices.

Future research can build on this foundational work in several important ways. For example, a more detailed comparison of classical and quantum algorithms for large network separation problems would be valuable. While the D-Wave hybrid solver has shown itself to be computationally fast, an exact comparison of computational times for very large networks (beyond 1492 nodes) could enhance future results and better inform decision-making. The model can be further evaluated by comparing predicted results to actual wildfire data. Furthermore, this study intends for the fuelbreaks to be made pre-emptively, when specific conditions such as wind and fuel moisture would be unknown. Not including these variables does make the model less accurate. Incorporating additional variables into future network models, such as wind speed, wind direction, and underlying vegetation composition, could lead to more precise and effective fuelbreak placement. Finally, future work could examine how discretizing the network impacts the final optimization. Addressing these, and other, open questions will help improve the results and refine the methods used. As uncontrolled fires have become an unavoidable threat, it is crucial to develop more robust approaches to forest fire management and preparedness. As catastrophic weather events continue, it is vital that the techniques we employ to tackle forest fires evolve to meet these growing challenges. This ongoing research is essential for creating more effective and lasting wildfire management strategies.

## Conclusion

Wildfires are becoming an increasingly urgent crisis, affected by human interaction such as land-use shifts and expanding settlements in fire-prone regions^26^. Effective fire mitigation strategies require the strategic placement of fuelbreaks, which serve as critical barriers to slow the spread of wildfires and protect ecosystems, infrastructure, and human communities. This study introduced an approach to fuelbreak optimization using network separation modeling and quantum computing, demonstrating that equal graph partitioning, when applied through D-Wave’s quantum hybrid solvers, offers a promising and efficient method for determining fuelbreak locations. By modeling wildfire spread as a network partitioning problem, our approach outperformed conventional heuristics, such as the ridgeline method, by achieving more effective land separation while minimizing the amount of land that needs to be cleared. In particular, the worst-case fire scenario analysis revealed that near-equal graph partitioning reduces the likelihood of large-scale forest loss while maintaining land conservation priorities.

Despite these promising results, we identify several key areas for future research. Incorporating realistic fire dynamics, including wind direction, fuel type, and topography, would substantially enhance the accuracy of this modeling approach. Since these environmental conditions play a crucial role in wildfire behavior, integrating real-time environmental data and machine learning models could provide a dynamic, adaptive optimization framework that adjusts fuelbreak placement based on evolving fire risk. The scalability of quantum computing for large-scale wildfire management also warrants further investigation, particularly in comparison to classical optimization solvers such as integer programming and metaheuristic methods. While quantum annealing has demonstrated advantages in handling large combinatorial problems, a systematic performance benchmark against traditional solvers is essential for validating computational benefits. Additionally, exploring hybrid quantum-classical approaches, such as variational quantum algorithms, could yield more refined and accurate solutions. Beyond computational improvements, a multi-criteria optimization framework could further enhance the practical utility of this approach. Future studies should evaluate how fuelbreak placement can balance ecological preservation, economic costs, firefighter accessibility, and community protection while maintaining fire containment effectiveness. Developing an interactive decision-support system for wildfire managers that integrates these factors would enable more strategic and adaptable planning. Furthermore, real-world validation of this method is crucial. Applying this approach to historical wildfire case studies and comparing predicted firebreak placements with actual fire spread patterns could provide valuable insights into its real-world applicability. Engaging fire management agencies to incorporate network-based optimization into operational decision-making would be a critical step toward practical implementation.

The rapid advancement of remote sensing, AI-driven fire forecasting, and geospatial analytics presents additional opportunities to integrate this approach with emerging technologies. Combining quantum-based firebreak optimization with real-time satellite data, drone imagery, and GIS-based fire modeling could lead to automated and highly responsive fire mitigation strategies. The broader implications of this research extend beyond wildfire management, as network science and quantum computing hold immense potential for addressing complex environmental and infrastructural challenges. As quantum hardware and algorithms continue to evolve, fire managers may soon have access to unprecedented computational power that enables real-time, adaptive firebreak planning. This could revolutionize wildfire mitigation efforts, transforming them into proactive, intelligent, and resource-efficient processes. Looking ahead, cross-disciplinary collaboration between fire ecologists, network scientists, AI researchers, and quantum computing experts will be essential to advancing scalable, data-driven wildfire prevention strategies. By fostering these partnerships and continually refining optimization models, this research has the potential to shape the future of wildfire management, offering new pathways for protecting landscapes, communities, and economies from the escalating dangers posed by wildfires. As wildfires become an unavoidable challenge, developing cutting-edge, computationally advanced strategies will be crucial in ensuring resilience against increasingly severe fire events.

## Data Availability

Forest geometry and land use data used for generating the network in this study are publicly available through OpenStreetMaps^17^, whose homepage is https://planet.osm.org. Elevation data for determining the maximum spotting distance is available publicly through the Elevation Point Query Service^18^ at https://epqs.nationalmap.gov. Data for creating the maximum spotting distance Eq. (1) is publicly available through the National Wildfire Coordinating Group^19^ at https://www.nwcg.gov/publications/pms437/crown-fire/spotting-fire-behavior.

## Code availability

Code for this project is publicly available at https://github.com/erdc/fire-separation^27^.
